# DC^2^Net: An Asian Soybean Rust Detection Model Based on Hyperspectral Imaging and Deep Learning

**DOI:** 10.34133/plantphenomics.0163

**Published:** 2024-04-05

**Authors:** Jiarui Feng, Shenghui Zhang, Zhaoyu Zhai, Hongfeng Yu, Huanliang Xu

**Affiliations:** ^1^ College of Artificial Intelligence, Nanjing Agricultural University, Nanjing, 210095, China.; ^2^ College of Engineering, Nanjing Agricultural University, Nanjing, 210095, China.

## Abstract

Asian soybean rust (ASR) is one of the major diseases that causes serious yield loss worldwide, even up to 80%. Early and accurate detection of ASR is critical to reduce economic losses. Hyperspectral imaging, combined with deep learning, has already been proved as a powerful tool to detect crop diseases. However, current deep learning models are limited to extract both spatial and spectral features in hyperspectral images due to the use of fixed geometric structure of the convolutional kernels, leading to the fact that the detection accuracy of current models remains further improvement. In this study, we proposed a deformable convolution and dilated convolution neural network (DC^2^Net) for the ASR detection. The deformable convolution module was used to extract the spatial features, while the dilated convolution module was applied to extract features from the spectral dimension. We also adopted the Shapley value and the channel attention methods to evaluate the importance of each wavelength during decision-making, thereby identifying the most contributing ones. The proposed DC^2^Net can realize early asymptomatic detection of ASR even when visual symptoms have not appeared. The results of the experiment showed that the detection performance of DC^2^Net dominated state-of-the-art methods, reaching an overall accuracy at 96.73%. Meanwhile, the experimental result suggested that the Shapley Additive exPlanations method was able to extract feature wavelengths correctly, thereby helping DC^2^Net achieve reasonable performance with less input data. The research result of this study could provide early warning of ASR outbreak in advance, even at the asymptomatic period.

## Introduction

Soybean [*Glycine max (L.) Merrell*] is an important food crop worldwide. It originates in China and accounts for 57% of the world's oil production, ranking first. However, since the 20th century, global production of soybean has suffered huge losses due to pests and diseases, with the most damaging one being Asian soybean rust (ASR) [1]. The causal agent of soybean rust, *Phakopsora pachyrhizi Syd*, can cause lesions on leaves, petioles, stems, and other parts [2]. Soybean infected with the rust disease, would usually encounter massive yield reduction, up to 50%. ASR can migrate long distances with air currents during the growing season [3] and is present in most soybean growing countries [4]. It is a major threat to soybean production in South America, Africa, and the United States [5], causing economic losses of 7 billion to 10 billion USD per year. It is also one of the most destructive and prominent diseases affecting soybean production in Brazil, with yield losses being 90% [6]. Furthermore, soybean rust has been documented in 23 provinces in China. Early detection of ASR has become an important and urgent task. Early detection can guide farmers to take timely and effective measures to control the spread of the disease and avoid huge economic losses [7].

The traditional method of detecting ASR by visual inspection by plant pathologists or soybean growers is time-consuming and inefficient. When the host interacts with the pathogen but does not cause visible disease symptoms, the soybean is in the incubation period, which is called asymptomatic infection [8]. It is not possible to determine whether a plant is healthy or asymptomatic by visual inspection or RGB images [9]. Most of the current mainstream disease diagnostic methods are carried out after the plant has developed obvious symptoms, making it difficult to detect and control the disease as early as possible. In recent years, with substantial advances in sensing technologies such as multispectral, hyperspectral, fluorescent, and thermal sensors, the detection of plant diseases through the use of such sensors has become trending [10]. In the early stages, when visual symptoms on the plant surface are absent, the pathogen still causes changes in internal physiological mechanisms, changes in cell structure, and production of toxic compounds [11]. These changes alter the spectral reflectance characteristics of plant leaves and provide a potential early indication of the disease infection process. Nagasubramanian et al. [12] used a genetic algorithm and support vector machine to select 6 wavelengths’ combinations from hyperspectral images in the range of 383 to 1,032 nm for early detection of soybean stem charcoal rot. The classification accuracy for infected categories was 97%. Hyperspectral sensors can obtain spectral information with hundreds of wavelengths at high resolution, thus allowing effective quantification and detection of changes in the physiological structure of plant leaves due to disease infestation that are often subtle and not detected by other sensors such as RGB cameras [13]. Therefore, hyperspectral sensors enable rapid, nondestructive early detection of crops and are one of the most promising methods for completing the plant disease detection task.

Nowadays, various studies have been conducted to complete the plant disease detection task. The development of hyperspectral imaging (HSI) and artificial intelligence offers new solutions. Deep learning technique provides a more promising solution compared with other traditional approaches, allowing for better decision-making [14]. The use of convolutional neural network (CNN) architectures demonstrates superior performance over traditional machine learning methods, because CNN is able to extract the most salient features from images [15]. Feng et al. [16] constructed a CNN model and experimentally demonstrated that the CNN model was better than traditional machine learning models for classification of rice hyperspectral data, and the accuracy of the test set reached over 93%. Yong et al. [17] trained the VGG16 model on raw images at a wavelength of 938 nm, achieving excellent performance with an accuracy of 91.93%, a precision of 94.32%, a recall of 89.26%, and an F1 score of 91.72%. The model successfully accomplished accurate classification of plant seedlings infected with basal stem rot. While hyperspectral data has been successfully used for disease detection, traditional machine learning methods still rely on time-consuming manual feature extraction. Cao et al. [9] proposed a method combining hyperspectral images and 3-dimensional (3D) convolutional neural network (3DCNN) to detect early asymptomatic infections of rice leaf blight and extracted 8 feature wavelengths that played a crucial role in the disease detection task. Rangarajan et al. [18] employed 8 different lightweight CNNs to efficiently predict wheat scab from canopy data. The DarkNet19 model achieved an F1 score of 100% based on spectral line graph images of smoothed and nonsmoothed data. Other deep learning models and algorithms used for detecting plant diseases include ResNet [19], GoogLeNet [20], VGG [21], AutoEnconder [22,23], and so on. These studies confirmed that spectral and spatial information from hyperspectral images combined with deep learning architectures have promising potential for crop disease detection [24–27].

However, a key issue with utilizing HSI is that the resulting hyperspectral data cubes comprised both spatial and spectral dimension, where the redundant information usually exists and might reduce the ability to distinguish between different object classes in the classification problem [28] Also, it was noted that the state-of-the-art (SOTA) disease detection models usually deployed standard 2-dimensional (2D) and 3D convolution kernels with fixed sizes. However, the disease spots of soybean rust are usually scattered in spots. Given the dynamics and uncertainty of disease outbreaks, infected areas in leaves vary in location, size, and shape. SOTA methods are limited in modeling such geometrical changes or transformations due to the fixed structure of the convolution kernel. It is not desirable for all convolutional units to have the same receptive field, especially when the objects to be detected are at different spatial locations and have different spectral responses.

Currently, in the progress of soybean rust research, it is only possible to rely on changes in external information such as leaf texture, color, spot shape, etc. to detect the disease [29] or the severity classification [10]. There lacks tools or methods that can detect crop diseases at an early asymptomatic stage of infection. To solve the above problems, this study introduced an early asymptomatic detection strategy based on 3DCNN by integrating deformable convolution and dilated convolution modules, enabling early disease detection and diagnosis of the most destructive soybean rusts. The main contributions of this study are summarized as follows:

1. An early soybean rust disease detection model, namely DC^2^Net (deformable convolution and dilated convolution neural network), was proposed by integrating the deformable convolutional and dilated convolutional modules. The deformable convolution replaced the standard 3D convolutional kernels for the purpose of extracting the spatial features of HSI data, while the 3D dilated convolution was added to extract the spectral features. The proposed DC^2^Net was able to capture informative features with various scales and shapes.

2. We applied the Shapley Additive exPlanations (SHAP) method to extract the feature wavelength. SHAP was able to evaluate the impact of a given spectral in comparison to the final output, i.e., the disease classification result. Also, the magnitude of SHAP value can be treated as a measure of how strong the effect would be. For detecting ASR, the most contributing wavelengths were successfully identified at the range of 580 to 650 nm by SHAP.

3. The ablation experiment was conducted to verify the effectiveness of both the deformable and dilated convolutional modules. Meanwhile, comparative evaluation was performed among DC^2^Net and SOTA models on the established ASR hyperspectral dataset, as well as a public dataset (Cassava Spectral Data).

We believe that the research result of this manuscript can also contribute to monitoring and managing diseases affecting other crops like rice and maize. The rest of this manuscript was organized as follows. Materials and Methods presented data collection and model establishment. Results described the experiment setup and results. Comparison with other research work and limitation of our proposal were discussed and the conclusion was drawn in Discussion.

## Materials and Methods

### Overall workflow

**Fig. 1.**
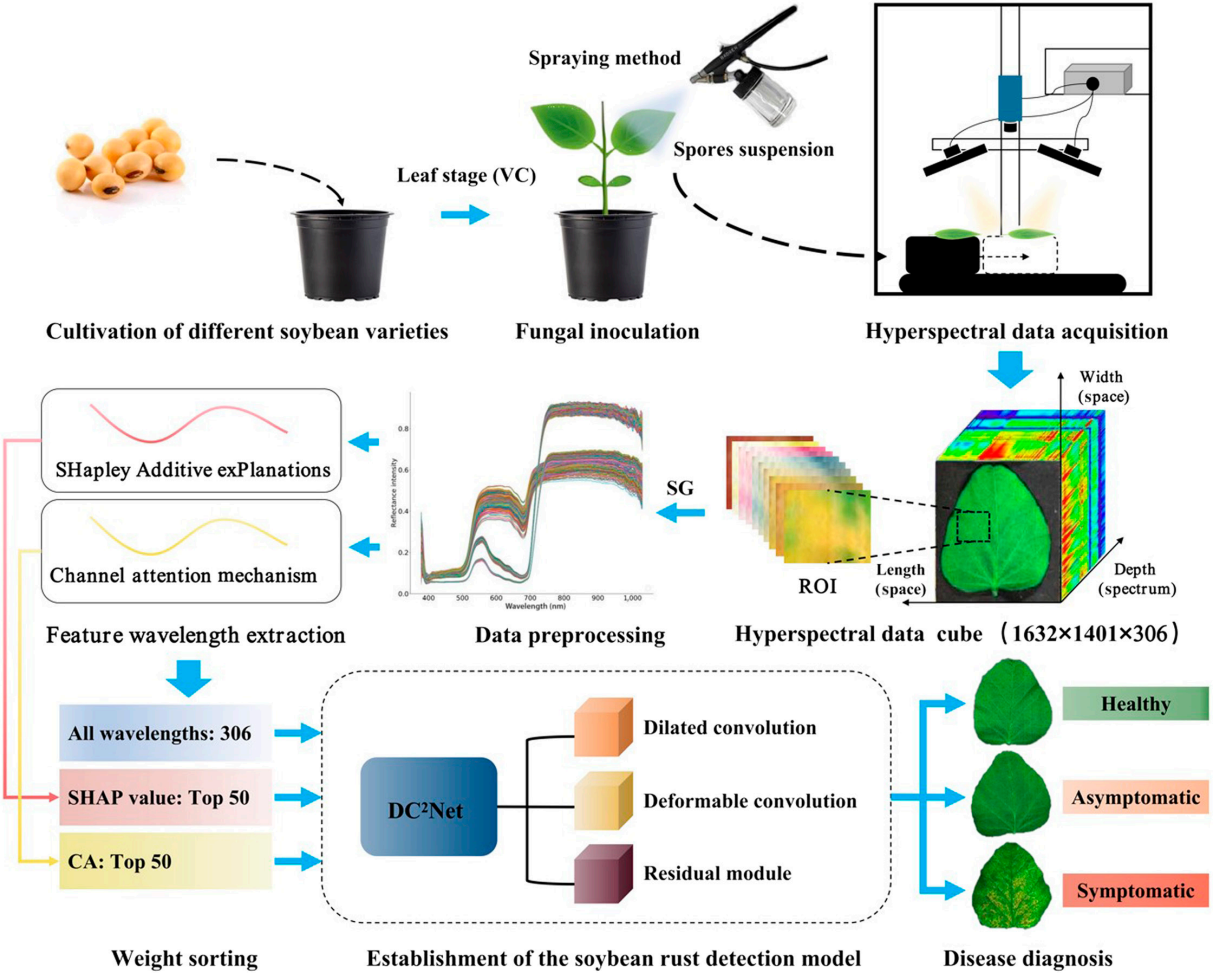
The overall workflow of this study.

The overall workflow designed for diagnosis of soybean rust was shown in Fig. 1. The process consisted of the following steps: (a) cultivation of soybean, (b) fungal inoculation, (c) hyperspectral data acquisition, (d) region of interest (ROI) extraction and data preprocessing, (e) feature wavelength extraction, (f) establishment of the soybean rust detection model, and (g) disease diagnosis.

### Plant materials

The experimental materials were cultured in March 2023 in a facility environment at Nanjing Agricultural University, Nanjing, Jiangsu Province, China. The soybean seeds (Table S1) were soaked, germinated, and sown into plastic pots with 3 holes and 1 seed per hole. The cotyledon stage was reached when the single leaf blade unfolded 10 d after soybean emergence. At this point, 2 well-grown soybean seedlings from each pot were retained for fungal inoculation. Subsequently, freshly collected soybean rust leaves were prepared into a spore suspension and inoculated onto soybean leaves at the 2-leaf stage by spraying. The leaves were incubated at 25 °C, 100% relative humidity, and darkness for 24 h and then placed in the facility environment. The incubation temperature was controlled between 25 and 27 °C, relative humidity was greater than 70%, and the light was illuminated for 14 h per day with an average light intensity of 5,000 Lx. The ratio of healthy and diseased plants was 1:2.

### Data acquisition and preprocessing

**Fig. 2.**
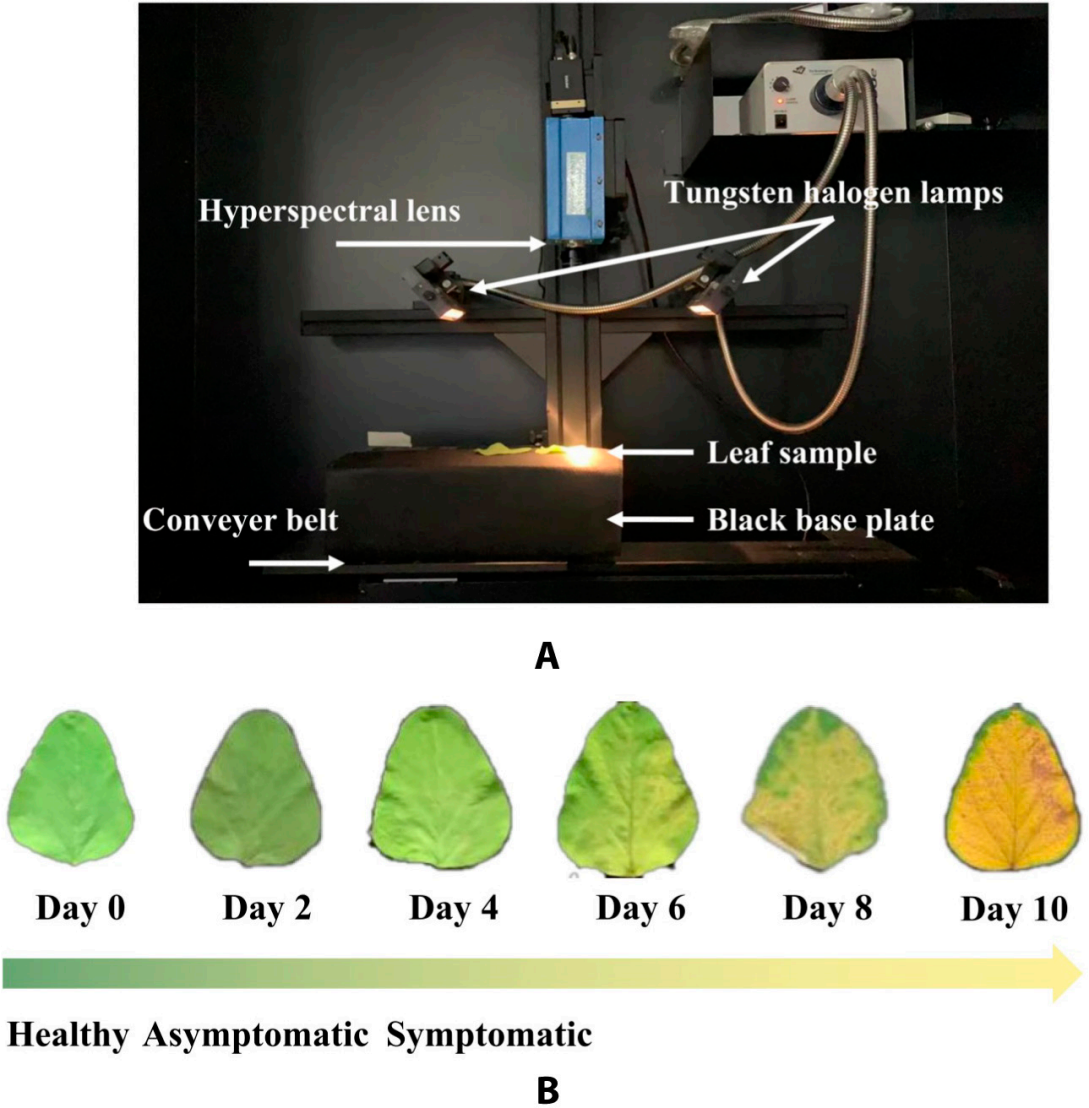
Hyperspectral data collection and preprocessing. (A) Schematic diagram of the HSI system. (B) Visual changes in soybean rust leaves.

This experiment was carried out using a push-scan HSI system (HSI-VNIR-0001, Shanghai Wuling Optoelectronics Technology Corp., China). The imaging system consisted of an imaging spectrometer (ImSpector V10E; Special Imaging Limited, Finland), a high-sensitivity electron-multiplying charge-coupled device camera (EM285CL, Raptor Photonics Ltd., United Kingdom), a long lens (OLES23, Special Imaging Limited, Finland), 2 tungsten halogen lamps (21-V/200-W light source, Lighting Technology Corp., America), and a conveyor belt driven by a stepper motor. The software system consisted of the Spectrol Image-V10E software and HSI Analyzer analysis software. The HSI system is shown in Fig. 2A.

The imaging system was warmed up for 5 min before collecting hyperspectral images. A 35-mm imaging lens was selected, the object distance was set at 27 cm, and the light source intensity was 100 Lx [9]. The sample leaves were laid flat on a black plate and placed on the conveyor platform vertically below the hyperspectral camera. The exposure time was adjusted by 8 ms to bring Max DN to 3,000 with an image resolution of 1,632 × 1,401. The blade was delivered to the ImSpector's field of view at a speed of 1.4 mm/s. The movement speed was chosen by trial and error to avoid distortion of image size and spatial resolution. The system collected spectral wavelengths ranging from 378.28 to 1,033.05 nm with a spectral resolution of 2.14 nm, containing a total of 306 wavelengths, and finally, we obtained a hyperspectral image data cube with the shape of 1,632 × 1,401 × 306. All acquired hyperspectral images were black and white corrected using Eq. 1:

$$R=\frac{R_{0}-D}{W-D}\times 100$$ (1)

where *R* is the calibration image, $R_{0}$ is the original hyperspectral image, *D* is the dark reference image, and *W* is the white reference image.

We built a dataset containing 660 hyperspectral images of soybean leaves, and ENVI 5.3 (Research System Inc, Boulder, CO, USA) software was used to analyze the raw hyperspectral images. Ten ROIs were randomly extracted for each leaf image after correction, resulting in 6,600 samples in total. Each plant was collected every 2 d until the leaves became severely diseased and fell off. We collected the hyperspectral data at fixed time (0900 to 1600) and adopted identical instrument parameters to ensure the consistency of the data. The labels were defined according to visual inspections by agronomy experts, with criteria as in Fig. 2B. Specifically, we defined soybean leaves that were inoculated with water as the healthy category and soybean leaves that were inoculated with the pathogens but no visible spots as the asymptomatic infection category. Similarly, soybean leaves inoculated with the pathogens but with visible spots as the symptomatic infection category. To eliminate the random noise of the spectral signal, we used the Savitzky–Golay (SG) smoothing filter for noise reduction [30].

Besides the smoothening operation, we also performed the feature wavelength extraction. The SHAP [31,32] and channel attention (CA) methods [33] were applied to evaluate the contribution of each wavelength to the prediction. On the one hand, extracting characteristics wavelengths enabled the model to generate outputs with fewer inputs, leading to the fact that the computational cost would be greatly reduced. On the other hand, feature wavelengths would help us unmask the black box (i.e., deep learning model) and interpret the decision-making mechanism of DC^2^Net.

### The proposed approach

In Architecture of DC^2^Net, we provided a comprehensive overview of the DC^2^Net model framework. Subsequently, in Dilated convolution and Deformable convolution, we introduced the applications of dilated convolution and deformable convolution in the detection of soybean rust. It is noted that soybean rust disease may exhibit complex morphological changes on the leaves, including irregular spot-like distributions. Due to the relatively limited capture of receptive fields by traditional 3DCNN, it becomes challenging to effectively encompass both global and local features of soybean rust in hyperspectral data. Therefore, the use of dilated convolutions, by introducing gaps between convolutional kernel elements, effectively enlarges the receptive field. 3D deformable convolution, by adaptively adjusting the sampling positions within the convolutional kernel, allows for more flexible capturing of local features, particularly when dealing with irregular shapes and variations in diseases.

When dealing with 3D hyperspectral data, a considerable number of computational resources is required, leading to a relatively lower computational efficiency. To overcome this challenge, our study integrated dilated convolutions and 3D deformable convolutions in place of standard convolutional kernels. These were applied separately in the spectral and spatial dimensions, enlarging the receptive field. This enhancement aimed to improve the model's sensitivity to early manifestations of diseases, thus enhancing the accuracy of disease detection.

#### Architecture of DC^2^Net

**Fig. 3.**
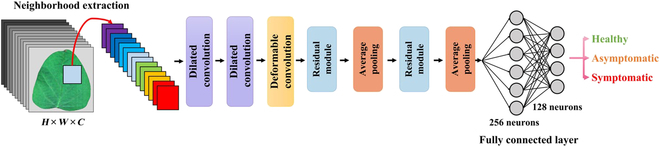
The architecture of the DC^2^Net model.

In this study, DC^2^Net was proposed for detection of ASR, and its architecture is shown in Fig. 3. DC^2^Net consisted of a deformable convolution module, 2 dilated convolution modules, 2 residual modules, 2 average pooling layers, and 2 fully connected layers.

The model first extracted the input data 25 × 25 × C (where C represents the number of hyperspectral channels) according to the ROI. Then, the feature information of the spectral dimension was extracted sequentially by 2 spectral dilation convolution modules, with 4 and 8 convolution kernels, respectively. The dilation convolution kernel had the shape of 3 × 3 × 5 size. The obtained feature maps were transferred to the deformable convolution module, and the receptive field of the convolution kernel was adaptively adjusted to capture the spatial information of the rust spots on soybean leaves. In this process, the size of the feature maps obtained by the deformable convolutional layer remained the same as the input from the previous layer. Meanwhile, in deep neural networks, the problems of gradient vanishing and gradient explosion would usually occur as the network deepens, in addition to increasing the consumption of computational resources and model overfitting problems, so we added 2 residual modules to the DC^2^Net network model and reduced the number of parameters by adding a 2 × 2 × 2 average pooling layer after the residual module. Finally, the classification task was accomplished by 2 fully connected layers with 256 and 128 neurons, respectively. The Softmax activation function generated the probability of each category as the output.

In the DC^2^Net, we first used spectral dilated convolution modules to extract features in the spectral dimension without adding additional parameters, and then we used the deformable convolution module to obtain an adaptive receptive field in the spatial dimension to extract the spatial features. The detailed hierarchical summary of DC^2^Net is shown in Table S2.

#### Dilated convolution

Standard convolution typically uses fixed-sized kernels, while dilated convolution kernels [34] can expand the receptive field by introducing a dilation rate (DR) without increasing the number of trainable parameters [35]. The dilated convolution can extract the feature information from raw spectral data by performing convolution operations on the spectral dimension [36].

**Fig. 4.**
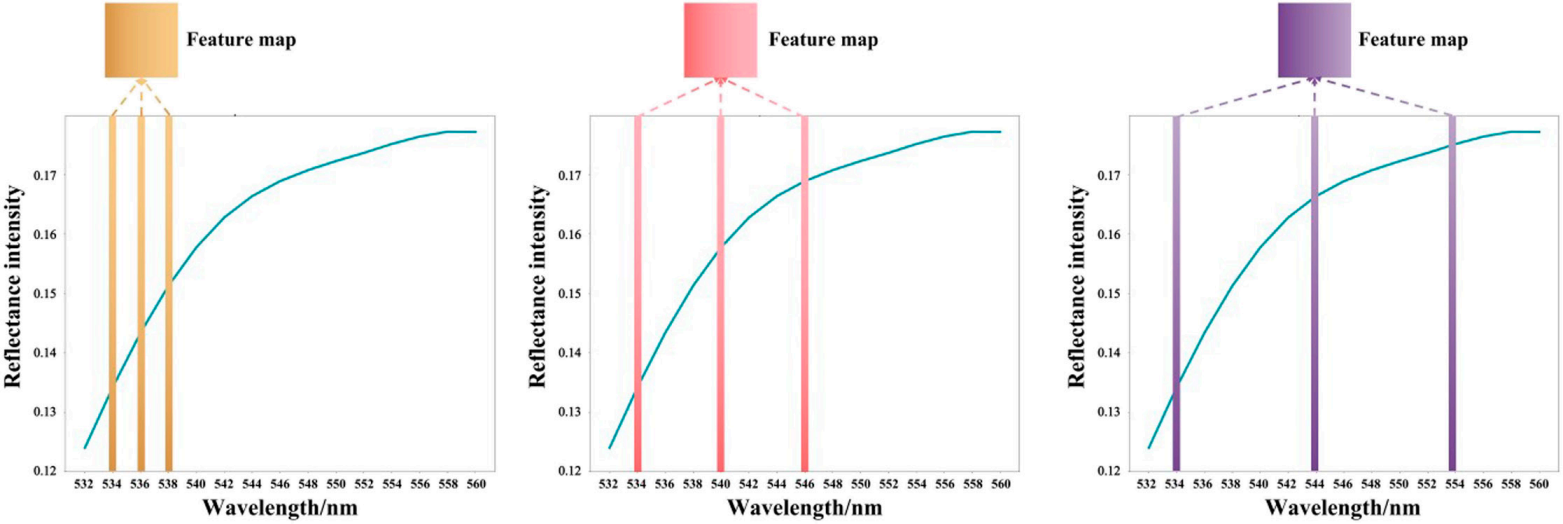
Schematic of the dilation convolution module. (A) DR = 0, (B) DR = 2, and (C) DR = 4.

As shown in Fig. 4, we took 15 wavelengths as an example. By increasing the DR (i.e., DR = 1, 2, 3, ...), dilated convolution extended the receptive field along the spectral dimension. In our previous research, we conducted comparative experiments with different DRs in the rice disease detection task [9]. In this study, we followed a similar approach to examine the ideal DR and reached the conclusion that when DR was set at 4, the DC^2^Net was able to achieve better performance. As ASR usually leads to a reduction in chlorophyll content in the leaf tissue, it produces troughs in the spectrum. The dilated convolution can enhance and highlight these trough features, and by dilating the receptive field, redundant information in neighboring wavelengths can be removed, leading to accurate classification.

#### Deformable convolution

Deformable convolution has achieved remarkable results in many image processing tasks, such as object detection and semantic segmentation [37] [38]. In the traditional convolution operation, the convolution kernel samples the input feature map at a fixed position to extract spatial information of the image, but this results in that all activation units in the same convolution layer have the same size of the receptive field, which is obviously undesirable for those objects with complex morphological structures (e.g., the spots of soybean rust leaves) [39]. Therefore, this study used deformable convolution to obtain adaptive receptive fields, so that the convolutional layer can capture long-range semantic information in the spatial dimension, which is more conducive to obtaining critical features in the feature map.

Deformable convolution V1 [40] (denoted by deform2Dv1 in the following) introduced a learnable offset in the computation of the feature map, which was used to represent the sampling offset position of the convolution kernel. Deformable convolution V2 [41] (denoted by deform2Dv2 in the following) added a modulation mechanism to the first version. This mechanism allowed each sample point to learn not only the offset but also the feature amplitude by the learned feature. In this study, we adopted the 3D deformable convolution [42] (denoted by deform3D in the following) to be implemented in a 3DCNN and applied it to the early detection of soybean rust.

In our study, we denoted the feature map of an image with resolution *H*×*W*×*C* by $X\;\in \;R^{H\times W\times C}$. Let *X(P)* denote the value of X at coordinates $P=\left(P_{x},P_{y},P_{z}\right)$. Also, denote W as the weight of a given convolution kernel containing K convolution points and b as the bias value of the current convolution layer. Let $D\in R^{K}$ denote the sampling region of the convolution. Suppose that for a standard convolution of size 3 × 3 × 3, the dilation ratio is 1, K = 27, and the sampling region is *D* = {(−1, −1, −1), (−1, −1, 0), …, (1, 1, 0), (1, 1, 1)}. In 3D deformable convolution, we used $\Delta D\in R^{K\times 3}$ to denote the offset of each point in the above sampling region. If we let Y denote the feature map obtained after applying the deformable convolution, then the value of Y at coordinate $P_{0}$ can be computed by Eq. 2:

$$\begin{array}{c} Y\left(P_{0}\right)=\sum_{p_{n}\in D}W\left(P_{n}\right)\cdot X\left(P_{0}+P_{n}+\Delta D\right)+b\; \end{array}$$ (2)

where $P_{n}$ enumerated the locations in the sampling region.

From Eq. 2, it can be seen that the variation of the convolution kernel feature extraction location was determined by learning an offset ΔD from the convolution kernel sampling location. Thus, deformable convolution can adaptively focus on spatial locations outside a fixed sampling region *D*, whereas traditional 3D convolution can only look at a fixed spatial location. Since the offsets obtained during training are usually fractional, this means that the position of each location in the convolution kernel after offset does not correspond to the actual pixel points present in the feature map. Therefore, it is necessary to use interpolation to determine the pixel values after the offset, which can usually be achieved by using trilinear interpolation, formulated in Eq. 3 as follows:

$$X\left(P\right)=\sum_{I}G\left(I,P\right)\cdot X\left(I\right)\;$$ (3)

where P denotes the actual position after offset $(P=P_{0}+$$P_{n}+\Delta D)$ for Eq. 2; I enumerate all positions in the feature map *X*. *G* denotes the trilinear interpolation. Trilinear interpolation is an interpolation method used to estimate the value at an arbitrary location based on the values of known data points in 3D space. First, the 8 data points near to the target position were found, which can form a cube in 3D space. The distances between the target position and that 8 points in 3 dimensions were calculated. Finally, based on the distance ratio, the values of these 8 data points were interpolated and calculated to obtain the value of the target position.

The core idea of using 3D deformable convolution on hyperspectral data is to first use 3D convolution to extract the features of hyperspectral data, and then dynamically learn the offsets of the generated feature maps in 3D space, and utilize these offsets to adaptively adjust the location of the features extracted by the convolution kernel. Finally, the true values of the sampling points are determined using trilinear interpolation, and the obtained true feature values are weighted and summed.

**Fig. 5.**
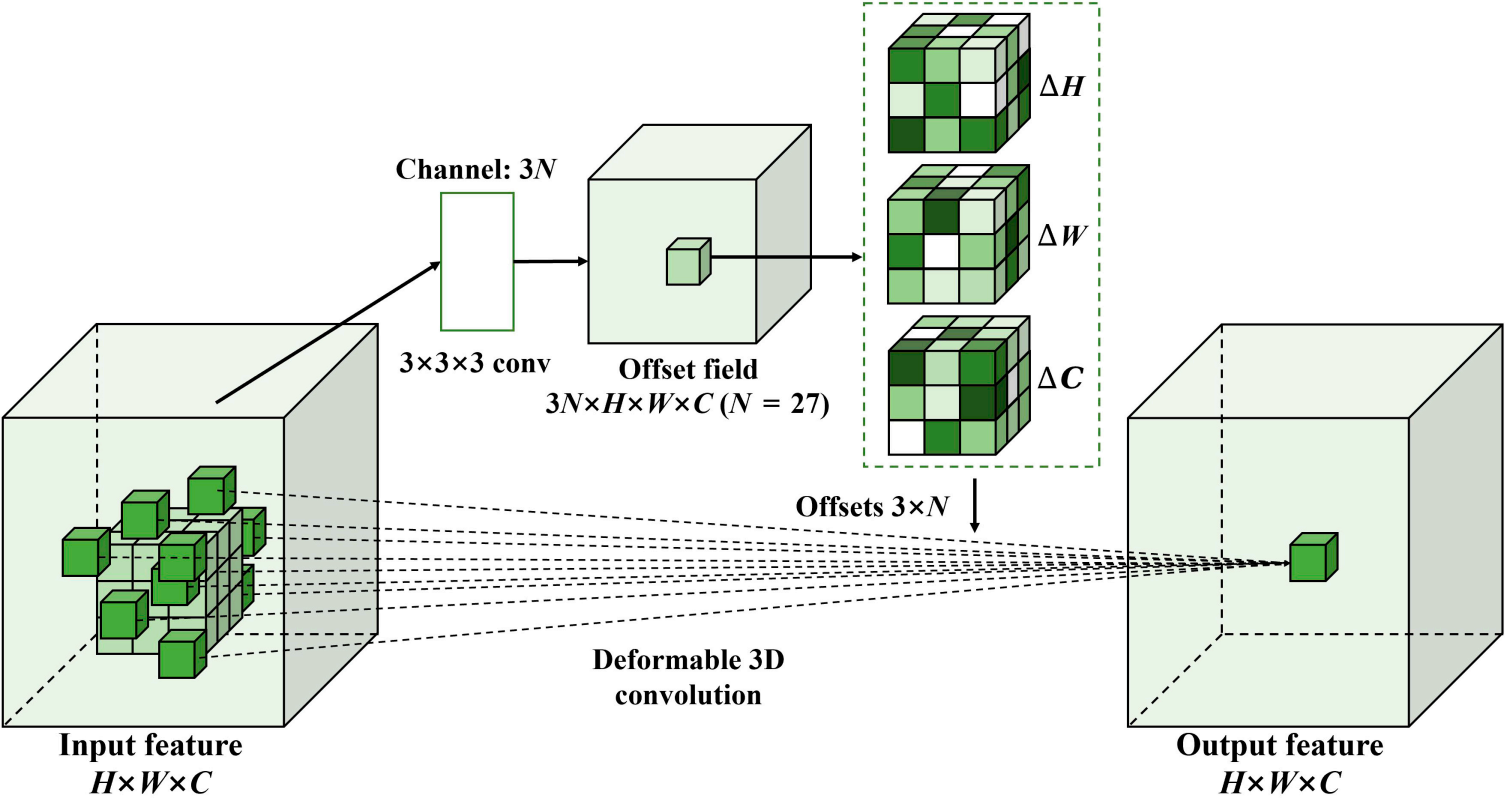
The flowchart of the 3D deformable convolution module.

In this study, we specifically focused on the application of deformable convolution modules to soybean rust hyperspectral data. This module can more flexibly capture local features by adaptively adjusting the sampling position within the convolution kernel. It had great advantages in dealing with morphological changes in soybean leaves and the irregularity of rust spots. Figure 5 shows the flow chart of the deformable convolution module implementation. In the input feature with dimensions *H*×*W*×*C*, the light green cube represents the sampling grid of a plain 3 × 3 × 3 convolution, and the dark green cubes represent the sampling grid of a deformable 3 × 3 × 3 convolution. We can learn the offsets of each feature map through the convolutional layer, so that the hyperspectral spot information contained in different locations were obtained. The offsets were generated by an offset generator (3 × 3 × 3 convolution), the channel size of the offset field was 3N, indicating that the convolutional kernel learned the offsets in the x, y, and z directions, respectively.

For the hyperspectral data of the soybean rust disease, each pixel contained information from multiple spectral bands. The key aspect of deformable convolution lied in its ability to adapt the receptive field for each position by allowing the convolutional kernel to adjust to spatial information at different locations. Compared to traditional fixed convolutional kernels, deformable convolutional modules enabled the model to have a more flexible perception of the local structure in the image. This adaptability enabled the model to more accurately capture subtle features and shape changes in the diseased areas of the hyperspectral image, particularly in response to variations in leaf morphology and irregularities in rust spots. This enhanced the model's ability to identify disease-related features.

#### Residual module

Hyperspectral images have high dimensionality, and it is well known that CNNs are improved mainly by widening or deepening the network. As the network deepens, the problems of gradient vanishing and gradient explosion occur, in addition to increased computational resource consumption and model overfitting problems. The Res-Net approach to this problem is "direct mapping", i.e., the next layer includes not only the information in that layer but also the new information in that layer after the nonlinear transformation [9]. Such processing makes the information will show an incremental trend layer by layer. Improved generalization performance of networks by introducing residual learning in deep network structures. In the residual module, the output of the lth block is calculated by Eq. 4:

$$x_{l+1}=F\left(x_{l}\right)+h\left(x_{l}\right)$$ (4)

where $x_{l}$ and $x_{l+1}$ are the input and output, respectively, of block *l*. Because the number of feature maps of $x_{l}$ and $x_{l+1}$ is the same, $h\left(x_{l}\right)$ is the identity map, that is, $h\left(x_{l}\right)=x_{l}$ which is reflected as the arc on the right side of Fig. S1. The residual module part included a skip connection and a main path. By stacking multiple residual modules, the network can gradually learn more complex and abstract feature representations.

### Experimental setup

#### Dataset description and training details

The hyperspectral data cube was represented by $\;I\;\in \;R^{H\times W\times C}$, where *I* denotes the original input, *H* is the height, *W* is the width, *H* and *W* denotes the spatial location information of the pixel in a 2D plane, and *C* denotes the number of spectral wavelengths. Each pixel in I has a label vector *Y* = (y_1_, y_2_, y_3_). In this study, we acquired the 6,600 hyperspectral soybean leaf samples and classified them into 3 categories: healthy, asymptomatic, and symptomatic.

Firstly, in order to pass the HSI cube into DC^2^Net, we divided the HSI data cube into small overlapping 3D spatial blocks, and the true value of each 3D block was determined by the label corresponding to the central pixel. We created data blocks of size *S*×*S*×*C* from the raw data to obtain a window of spatial extent *S*×*S* size and the spectral wavelengths after processing. The total number of 3D blocks generated from the raw hyperspectral data was calculated by (*H*−*S*+1) × (**W**−*S*+1). This process created a data block $P\;\in \;R^{S\times S\times C}$ centered at spatial position (a, b, c) covering the *S*×*S* spatial window. We set the acquired dataset into training set, a validation set and a test set with the ratio of 8:1:1. The percentage of data samples for each class in the dataset is shown in Table S3.

All experiments were conducted on the NVIDIA RTX A2000 GPU and 16GB RAM. The DC^2^Net model used cross-entropy as the loss function and used the stochastic gradient descent optimizer for training. We selected the optimal learning rate of 0.001, the weight decay coefficient was set to 1 × 10^-6^, dropout was set to 0.45, and batch size was set to 32, and we performed a total of 30 epochs for training [35].

#### Evaluating indicator

In this study, we calculated the overall accuracy (OA), average accuracy (AA), and kappa coefficient (Kappa) evaluation metrics to evaluate the HSI classification performance, based on the confusion matrix from the number of true positives (TP), true negatives (TN), false positives (FP), and false negatives (FN) [43]. The proportion of accurately classified positives among all identifications is called precision. Recall is the proportion of accurately identified positives among all true positives. On the other hand, the F1 score combines the accuracy and recall of the classifier by incorporating the harmonic mean of the classifier into a single metric. These metrics are given between Eqs. 5 and 11

$$\text{Precision}=\frac{TP}{\left(TP+FP\right)}$$ (5)

$$\text{Recall}=\frac{\mathrm{TP}}{\mathrm{TP}+\mathrm{FN}}$$ (6)

$$F1-score=\frac{2\times Precision\times Recall}{Precision+Recall}$$ (7)

$$OA=\frac{\left(TP+TN\right)}{\left(TP+FP+TN+FN\right)}$$ (8)

$$AA=\left(\frac{TP}{TP+FN}+\frac{TN}{FP+TN}\right)\times \frac{1}{2}$$ (9)

$$P_{e}=\frac{\left(TP+FN\right)\times \left(TP+FP\right)+\left(FP+TN\right)\times \left(FN+TN\right)}{{\left(TP+FP+TN+FN\right)}^{2}}$$ (10)

$$\text{Kappa}=\frac{OA-P_{e}}{1-P_{e}}$$ (11)

## Results

### Spectral analysis of soybean rust leaves

**Fig. 6.**
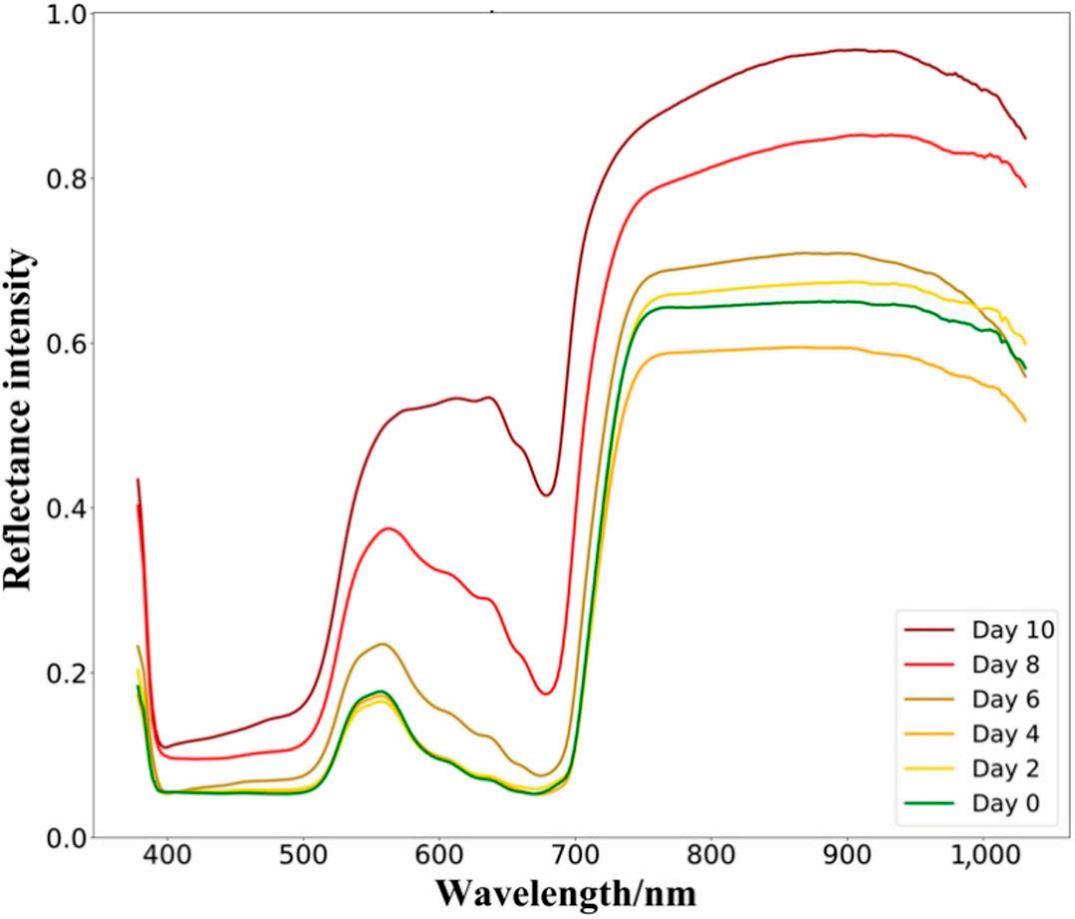
The average spectra of soybean leave with time after inoculation.

By monitoring the average spectra of soybean rust leaves, we can observe the trend over time, as illustrated by the specific spectral curves in Fig. 6. These curves exhibited notable differences, particularly in the wavelength ranges of 500 to 700 nm and 750 to 950 nm. The prominent differences in these wavelength ranges may reflect the biochemical and optical changes induced by soybean rust disease within these specific spectral regions. Through a thorough analysis of these differences, we can gain a more comprehensive understanding of the development and pathological processes of soybean rust disease. This provided robust spectral support for further disease detection efforts.

### Performance comparison of deformable convolutional modules

To evaluate the effectiveness of the 3D deformable module, we first compared the 3D deformable convolution modules (denoted by deform3D in the following experiment) [42] with 2D ones, i.e., the first version of the 2D deformable convolution module (denoted by deform2Dv1) [40] and its improved version (denoted by deform2Dv2) [41]. The full wavelengths (306 in total) were used as the input. Figure S2 showed the confusion matrices when 3D deformable convolution modules and two 2D ones being adopted in the DC^2^Net model. From the evaluation metrics in Fig. S2 and Table 1, it can be found that the 3D deformable convolution achieved the optimal results in all categories. Also, we noted that the performance of deform2Dv1 and deform2Dv2 had minor difference. Although the detection accuracy in the asymptomatic category was the lowest, deform3D greatly enhanced the classification performance in this category compared with deform2D. Deform3D achieved the highest precision in the symptomatic category, at 98.1386%. Combining the average results across all 3 classes, we can conclude that deform3D exhibited the best performance.

**Table 1.** Performance evaluation of the deformable convolution modules. Note: The values in bold font indicated the optimal result.

Class	Evaluation indicator	Models		
deform2Dv1		deform2Dv2	deform3D
Healthy	Precision	94.3840	95.0454	**95.7714**
Recall	94.7275	95.0454	**96.7727**
F1 score	94.5554	95.0454	**96.2694**
Asymptomatic	Precision	92.4283	92.6437	**94.9730**
Recall	93.7727	94.4545	**96.1818**
F1 score	93.0956	93.5403	**95.5735**
Symptomatic	Precision	96.0648	96.4765	**98.1386**
Recall	94.3181	94.5909	**95.8636**
F1 score	95.1834	95.5243	**96.9877**

### Ablation experiment

In order to evaluate the newly added modules in DC^2^Net, we further validated the impact of the deformable convolution and spectral dilation convolution modules on the underlying 3DCNN architecture through ablation experiments. Specifically, we compared the performance of the base 3DCNN architecture with and without the dilated convolution and deformable convolution modules. First, we introduced the dilated convolution module into the base 3DCNN to assess the impact of expanding the receptive field in the spectral dimension on the early detection of soybean rust. Next, we replaced the dilated convolution with the deformable convolution module to assess whether expanding the receptive field in the spatial dimension contributes to enhancing the accuracy of disease detection. Finally, we integrated both convolution modules to validate the effectiveness of the proposed DC^2^Net. Through ablation experiments, our aim was to gain a comprehensive understanding of the contribution of the added modules to the overall model performance.

Changes in leaf biochemistry due to disease stress would alter the spectral response of leaves, while changes in leaf morphological structure (e.g., wilting, curling) and texture can also affect the spatial information of leaves. Therefore, ablation experiments were conducted to test the effect of deformable convolution and dilated convolution on the accuracy of the early detection model for soybean rust, respectively. The evaluation results of the ablation experiments are presented in detail in Table 2. The dilated convolutional module improved the prediction accuracy by 2.5139%, 0.5275%, and 1.6866% for healthy, asymptomatic, and symptomatic categories, respectively. Dilated convolution, by adjusting the sampling positions of the convolution kernel, could enlarge the receptive field while keeping the kernel size unchanged, thereby improving the model's generalization capability. The deformable convolutional module improved the prediction accuracy by 10.7352%, 0.6540%, and 4.5664% for healthy, asymptomatic, and symptomatic categories, respectively. The deformable convolution module effectively adjusted the receptive field, and it can better be adapted to spatial variations. The experiments showed that the deformable convolution module had promising advantages for the early symptomless detection of the soybean rust disease. The Precision and F1 score of the health category, as well as the Recall of the asymptomatic category, have increased from 80% to over 90%. Further, the simultaneous addition of the dilated convolution and deform3D convolution enabled DC^2^Net to achieve the best performance, with accuracy, recall, and F1 score, reaching over 95%.

**Table 2.** Experimental result of the ablation evaluation. Note: The values in bold font indicated the optimal result.

Class	Evaluation indicator	Models			
3DCNN		3DCNN+dilated	3DCNN+deform3D	3DCNN+dilated+deform3D
Healthy	Precision	84.1741	86.6880	94.9093	**95.7714**
Recall	92.0000	93.3636	94.8977	**96.7727**
F1 score	87.9132	89.9020	94.9034	**96.2694**
Asymptomatic	Precision	94.2646	94.7921	94.9186	**94.9730**
Recall	83.8750	86.1477	95.7500	**96.1818**
F1 score	88.7668	90.2634	95.3324	**95.5735**
Symptomatic	Precision	91.6369	93.3235	96.2033	**98.1386**
Recall	93.2500	93.7614	94.3750	**95.8636**
F1 score	92.4364	93.5419	95.2803	**96.9877**

### Comparative experiment on feature wavelength extraction methods

The full wavelength of hyperspectral data has redundant and noisy information that can interfere with the predictive output of the model. Therefore, extracting the characteristic wavelengths as inputs to the model can, on the one hand, reduce the cost of data collection and, on the other hand, improve the prediction accuracy of the model. The above experimental results showed that the DC^2^Net proposed in this study achieved the best model performance, and in this section, we further validated the feature selection method. We used the first 50 wavelengths screened by the SHAP method and the CA method, as well as the full wavelengths as input. The effectiveness of the feature wavelength selection method used in this study was evaluated in Table S4. The SHAP method achieved first place in all evaluation metrics, both exceeding 96%.

**Fig. 7.**
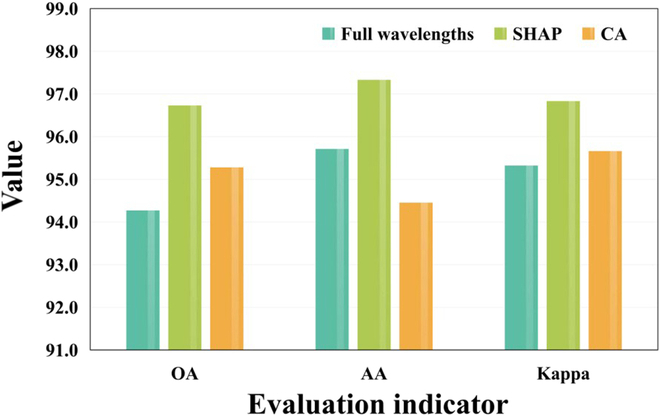
Overall performance of feature wavelength extraction.

In this study, OA (%), AA (%), and kappa coefficient (Kappa) were also chosen as classification evaluation indicators. Here, OA denotes the number of correctly categorized samples as a proportion of the total number of samples, AA denotes the classification accuracy for each category, and the kappa coefficient measures the agreement between the prediction and the ground truth. The evaluation results were shown in Fig. 7 where the overall performance of the SHAP method, represented by the green bars, dominated the other 2 methods. The features extracted by SHAP as inputs gave the best results in DC^2^Net model. OA was 96.73%, AA was 97.33%, Kappa coefficient was 96.83%. In addition, the experimental results suggested that the SHAP method would be more effective to extract feature wavelengths for detecting ASR.

We visualized the importance of all wavelengths by a heat map (Fig. S3). The characteristic wavelengths screened by 2 methods were concentrated in the 580- to 650-nm interval and around 700 nm. The bar chart in Fig. S4 showed the SHAP value values of the top 20 wavelengths that have the greatest impact on the prediction output generated by DC^2^Net, where each bar indicated the importance of a wavelength. Green, yellow, and red colors represented healthy, symptomatic, and asymptomatic categories, respectively. We can see that the top 4 ranked feature wavelengths (593, 583, 570, and 602 nm) were the most contributors to classify the symptomatic category. It was worth noting that the 690 nm has the smallest SHAP value in the symptomatic category, but 690 nm still had a major impact on identify the healthy and asymptomatic classes. We summed the SHAP values of all features for all samples, and this result reflected the feature importance and the contribution of each feature to the positive and negative predictions of the samples.

For more details, we presented the Bee swarm plot of the top 20 feature wavelengths for each category in Fig. S5. We found that the wavelengths with higher SHAP value were concentrated between 550nm-650nm which was between the yellow-green and orange-red light ranges. Yellow-green and orange-red light is one of the spectral ranges required by plants for photosynthesis. Healthy plant leaves are able to absorb these wavelengths better for photosynthesis, converting light energy into chemical energy for plant growth and development. The experiment proved that the performance of soybean leaves in the yellow-green and orange-red light ranges was more seriously affected under disease stress, indicating that ASR affects the chlorophyll content of soybean leaves at the early stage of infestation, alters the internal cellular structure, and reduces the light energy capture and nutrient uptake capacity of soybean leaves.

### Comparison of SOTA models

We also compared the proposed DC^2^Net with SOTA models used in HSI classification tasks, including HybridSN [44], Fast-3DCNN [45], SDC-3DCNN [9], MSR-3DCNN [46], HS-CNN [47], and Res-Net [47]. Based on the previous experiments, the comparison with the SOTA models all took the top 50 contributing feature wavelengths extracted by SHAP as inputs. In Table 3, the different models all achieved good classification results. The DC^2^Net model achieved the best results among all models. The proposed model achieved 97.7708% in the healthy category, 96.8732% in the asymptomatic category, and 97.7726% in the symptomatic category. The accuracy and loss plots of all the models were shown in Figure S6.

**Table 3.** Comparison of SOTA models on the ASR dataset. Note: The values in bold font indicated the optimal result.

Class	Evaluation indicator	Models						
Fast-3DCNN		HybridSN	SDC-3DCNN	MSR-3DCNN	HS-CNN	Res-Net	DC^2^Net
Healthy	Precision	93.2156	91.0951	94.2032	95.1756	90.3597	89.2375	**97.7708**
Recall	92.3409	94.7841	93.7614	94.8409	91.2752	88.2612	**96.9205**
F1 score	92.7761	92.9029	93.9817	95.0075	90.8151	88.7466	**97.3437**
Asymptomatic	Precision	85.3420	86.8768	94.9511	86.6217	92.3604	87.1024	**96.8732**
Recall	93.8750	92.3409	94.7841	96.6023	92.9908	88.9376	**96.7159**
F1 score	89.4053	89.5255	94.8675	91.3401	92.6745	88.0104	**96.7944**
Symptomatic	Precision	93.5131	95.2323	93.5556	96.2994	93.1478	88.4565	**97.7726**
Recall	85.0682	85.4091	94.1591	85.5795	92.2506	86.9372	**96.5978**
F1 score	89.0909	90.0536	93.8563	90.6235	92.6970	87.6902	**97.1816**

We utilized the cassava disease detection dataset from Owomugisha et al. [48], the Cassava Spectral Data. The dataset was collected using a C1-710 miniature leaf spectrometer with a wavelength from 345 to 1,043 nm. We selected a total of 306 bands within the 450- to 900-nm range following the same experimental procedure and employed the same strategy for ROI extraction. These samples were evenly categorized into 3 classes: Healthy (labeled as “0”), cassava mosaic disease (CMD (labeled as “1”), and cassava brown streak disease (labeled as “2”) [49].

Additionally, this publicly available dataset was utilized to validate DC^2^Net and all SOTA models, with detailed results presented in Table S5. The proposed DC^2^Net achieved the highest accuracy among all models and classifications. The recall of cassava brown streak disease was the lowest at 93.4204%, and the classification accuracy of other categories is above 94%. We also visualized the loss and accuracy graphs of the author dataset and the public dataset in all models, as shown in Figs. S6 and S7.

## Discussion

## The efficiency of different convolution kernels in feature extraction

Plant diseases usually have irregular shapes and are difficult to detect with the visual symptoms in the early stages. The limitations of visual inspection have stimulated interest in developing spectral-based methods for disease diagnosis [50]. Hyperspectral data are usually stereoscopic and contain both spectral and spatial information. Usually, deep learning models are designed to include different types of convolutional layers to capture various features at different levels. Standard convolution is commonly used to capture local features on the spectrum, such as peaks. 3D convolution can effectively capture the spatial correlation of images or data and preserve and utilize more 3D information when processing images, thus improving the model's ability to understand the content of the image, making it suitable for processing hyperspectral data with multiple channels. It helps in detecting changes in the characteristics of plant spots in the spectral dimension. The DC^2^Net model proposed in this study used a combination of multiple convolution forms (dilated convolution and deformable convolution) when performing disease detection on hyperspectral data to make full use of both spectral and spatial information. Among them, dilated convolution expanded the receptive field of the convolution kernel compared with the standard conventional convolution, which helped to capture a wider range of spectral dimension information. In addition, deformable convolution enhanced the adaptability of the model to irregular disease features and determines the spatial information of the disease more accurately. Although deform2D had achieved good classification results in hyperspectral data [51]. However, the experimental results of this study showed that the 3Ddeform convolution outperformed the original convolution and 2D deformable convolution for classification. This indicated that 3D deformable convolution is better at extracting spectral latitude and spatial deformable information. The comprehensive use of multiple convolution forms can help improve the performance and feature extraction ability of the model.

## The effectiveness of feature wavelength extraction

The characteristic wavelengths extracted in this study were similar to the results of the optimal wavelengths for soybean rust screened in the literature [10] and have the same spectral curves. Among the feature extraction methods selected in this paper, SHAP had good performance and can be applied to other hyperspectral fields for sensitive wavelengths extraction. In addition, the subset of wavelengths selected by the SHAP and CA methods in this paper was better than the original hyperspectral raw data (without feature wavelength extraction). This phenomenon can be explained that for raw hyperspectral data, the full wavelengths contained redundant and noisy information, which was harmful to the decision-making of DC^2^Net. The feature wavelength extraction removed these less informative wavelengths and retained the most contributing ones. This not only compressed the data and increased the training efficiency but also improved the performance of predictive analyses [33].

In the future, we can further explore the correlation between the characteristic spectral changes and the physiological changes in crops under different levels of stress. For example, additional experimental variables can be added to explore spectral differences, such as water stress, nutrient stress, disease stress, etc.

## Variety resistance detection

The disease detection model DC^2^Net proposed in this study was successfully used for early detection of soybean rust. However, disease occurrence evolves with time, and when monitoring individual plants or assessing the presence of disease in the field, the signals measured will vary in multiple conditions. For example, Abdelghafour et al. [52] tested 7 potato varieties, 6 of which showed detectable black spots, 36 h after inoculation. Plant–pathogen interaction is a dynamic process, and even healthy plants are subject to uncontrolled natural physiological fluctuations. Spectral data obtained at different growth stages may differ, so that changes in the temporal and spatial dimensions of the disease can be further captured in the future.

Early detection of plant diseases using automated, nondestructive, and high-throughput technologies is a major goal in plant breeding and crop protection. Combining spectroscopy with deep learning has great potential to not only directly assess disease severity but also to mine spectral features for rapid screening of disease-resistant varieties in high-throughput phenotyping [53]. Therefore, in future studies, we will perform calculations for different soybean varieties for disease resistance breeding. By combining high-throughput data collection, data management, data processing, and phenotype extraction [54], we can perform plant phenotyping analysis and variety resistance examination.

This paper presented DC^2^Net for early asymptomatic detection of ASR. We replaced the standard convolution kernel with the deformable convolution and dilation convolution. The disease detection model integrated 2 types of DC convolution modules, which can change the receptive field in spatial and spectral dimensions, respectively. Ablation experiments were performed to validate the effectiveness of deformable and dilated convolutional modules. The experimental results showed that DC^2^Net had an accuracy of 96.8732% in the detection of early asymptomatic stages of ASR and 97.7708% and 97.7726% in healthy and symptomatic detection, respectively. The model performance was optimal in several evaluation criteria compared with the SOTA methods. We also applied the SHAP method to extract the characteristic wavelengths and successfully identified the wavelengths with the highest contribution in the range of 580 to 650 nm. The results showed that the SHAP method can extract representative and interpretable wavelengths. This work validated the advantages of deformable convolution and dilated convolution modules in feature extraction of crop disease hyperspectral data, and provided a new solution for the application of HSI techniques and deep learning methods to identify early crop leaf diseases. In summary, the proposed model, DC^2^Net, would be a promising disease detection method, providing early warning of disease outbreak, and helps to improve the efficiency, accuracy, and sustainability of agricultural production.

## Data Availability

The data used in this study are available from the corresponding author upon reasonable request. The code of our work can be found via the following link: https://github.com/NJAUJerry/DC2Net.
