# Supplementary material for: DC2Net: An Asian Soybean Rust Detection Model Based on Hyperspectral Imaging and Deep Learning
Source: Plant Phenomics. 2024 Apr 5;6:0163. doi: 10.34133/plantphenomics.0163 (PMC10997487; doi:10.34133/plantphenomics.0163)
Supplement: Supplementary 1 — Figs. S1 to S7 Tables S1 to S5 [file plantphenomics.0163.f1.docx]

**Supplementary figures and tables**

**Table S1**. Information about 8 soybean varieties

| **Variety** | **Approval number** | **Applicable range** |
| --- | --- | --- |
| Zhonghuang 57 | National 2010005 | Shandong, Henan, Shanxi |
| Huaixian 5 | Su 201501 | Jiangsu |
| Tiedou 39 | Liao [2006] 84 | Liaoning |
| Dandou 21 | Liao 20190016 | Liaoning |
| Fudou 9 | Liao 20210002 | Lia ning |
| Liaodou 15 | National 2003010 | Xinjiang, Gansu, Ningxia, Shanxi |
| Jingxian 6 | — | Jiangsu |
| Jingxian 208 | — | Jiangsu |

**Table S2**. The network parameters of DC^2^Net

| **Layer(type)** | **Output Shape** | **Parameter** |
| --- | --- | --- |
| input_1 (InputLayer) | (25,25,50) | 0 |
| dilated_conv_1(Conv3D) | (23,23,44,16) | 448 |
| dilated_conv_2 (Conv3D) | (21,21,38,1) | 433 |
| deformable_conv | (21,21,38,1) | 2187 |
| conv3d_1 (Conv3D) | (21,21,38,8) | 16 |
| conv3d_2 (Conv3D) | (21,21,38,16) | 12688 |
| conv3d_3 (Conv3D) | (21,21,38,32) | 32 |
| conv3d_4 (Conv3D) | (21,21,38,32) | 544 |
| add_1 (Add) | (21,21,38,32) | 0 |
| pooling3d_1 (AveragePooling3D) | (10,10,19,32) | 0 |
| conv3d_5 (Conv3D) | (10,10,19,32) | 1056 |
| conv3d_6 (Conv3D) | (10,10,19,64) | 55360 |
| conv3d_7 (Conv3D) | (10,10,19,128) | 8320 |
| conv3d_8 (Conv3D)  add_2 (Add) | (10,10,19,128)  (10,10,19,128) | 4224  0 |
| pooling3d_2 (AveragePooling3D) | (5,5,9,128) | 0 |
| flatten_1 (Flatten) | (28800) | 0 |
| dense_1 (Dense) | (256) | 7373056 |
| dropout_1 (Dropout) | (256) | 0 |
| dense_2 (Dense) | (128) | 32896 |
| dropout_2 (Dropout) | (128) | 0 |
| dense_3 (Dense) | (3) | 387 |
| Total Trainable Parameters: 7,492,620 | | |

**Table S3**. Percentage of data samples for each class for dataset

| **Class** | **Train set** | **Validation set** | **Test set** |
| --- | --- | --- | --- |
| Total | 5280 | 660 | 660 |
| Healthy | 1690 (32%) | 211 (32%) | 211 (32%) |
| Asymptomatic | 1848 (35%) | 231 (35%) | 231 (35%) |
| Symptomatic | 1742 (33%) | 218 (33%) | 218 (33%) |

**Table S4.** Performance evaluation of DC2Net models using different input feature wavelengths

| **Class** | **Evaluation indicator** | **Models** | | |
| --- | --- | --- | --- | --- |
|  |  | **Full wavelengths** | **SHAP** | **CA** |
| Healthy | Precision | 95.7714 | **97.7708** | 96.9422 |
|  | Recall | 96.7727 | **96.9205** | 95.1818 |
|  | F1-score | 96.2694 | **97.3437** | 96.0539 |
| Asymptomatic | Precision | 94.9730 | **96.8732** | 96.7716 |
|  | Recall | 96.1818 | **96.7159** | 95.2248 |
|  | F1-score | 95.5735 | **96.7944** | 95.4974 |
| Symptomatic | Precision | 98.1386 | **97.7726** | 97.7153 |
|  | Recall | 95.8636 | **96.5978** | 96.4886 |
|  | F1-score | 96.9877 | **97.1816** | 97.0980 |

**Note:** The values in bold font indicated the optimal result.

**Table S5.** Comparison of SOTA models on the Cassava Spectral Data

| **Class** | **Evaluation indicator** | **Models** | | | | | | |
| --- | --- | --- | --- | --- | --- | --- | --- | --- |
|  |  | **Fast-**  **3DCNN** | **HybridSN** | **SDC-**  **3DCNN** | **MSR-**  **3DCNN** | **HS-CNN** | **ResNet** | **DC^2^Net** |
| Healthy | Precision | 91.3679 | 89.4838 | 92.5343 | 93.5353 | 88.4273 | 84.2453 | **95.4353** |
|  | Recall | 90.2572 | 92.7324 | 91.7404 | 92.3689 | 89.1208 | 82.2354 | **94.5404** |
|  | F1-score | 90.8091 | 91.0791 | 92.1356 | 92.9484 | 88.7726 | 83.2282 | **94.9857** |
| CMD | Precision | 88.3078 | 87.5786 | 91.8667 | 92.4210 | 90.2878 | 85.1035 | **95.5334** |
|  | Recall | 89.8875 | 85.3334 | 92.3412 | 94.2434 | 89.7657 | 83.8674 | **94.7543** |
|  | F1-score | 89.0906 | 86.4414 | 92.1033 | 93.3233 | 90.0259 | 84.4809 | **95.1422** |
| CBSD | Precision | 90.7563 | 92.2807 | 93.8674 | 93.2402 | 91.1827 | 82.3784 | **95.4541** |
|  | Recall | 87.0831 | 89.4838 | 94.4204 | 92.7557 | 89.8274 | 83.3341 | **93.4204** |
|  | F1-score | 88.8817 | 90.8607 | 94.1430 | 92.9973 | 90.4999 | 82.8534 | **94.4263** |

**Note:** The values in bold font indicated the optimal result.

| 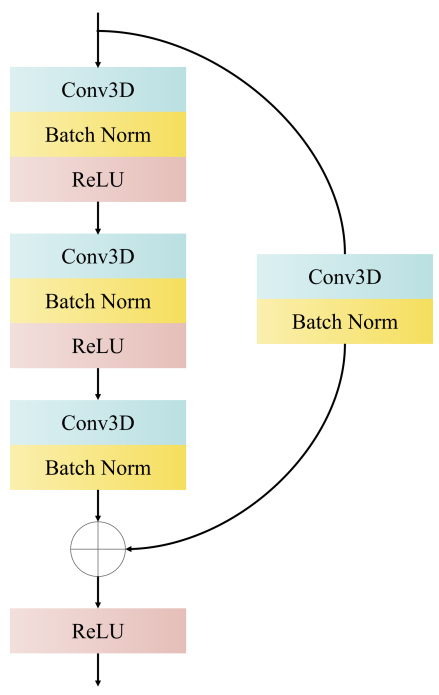 |
| --- |

**Figure S1.** Architecture of the residual block. **Note:** The size of the convolutional kernel in the skip connection was $\text{1×1×1}$. The skip connection helped the information to be passed directly to the subsequent layers, thereby avoiding the loss or attenuation of the information. The main path contained three 3D convolutional kernels with shape of $\text{1×1×1}$, $\text{3×3×3}$, and $\text{1×1×1}$, respectively, which are mainly responsible for learning the residual mapping. A batch normalization layer and a ReLU activation function were also added after the convolutional layer. The batch normalization layer was mainly used for data normalization and parameter scaling to accelerate network training and improve generalization, while ReLU introduced nonlinear transformations and sparsity to help the network learn complex features and alleviate the gradient vanishing problem.

| 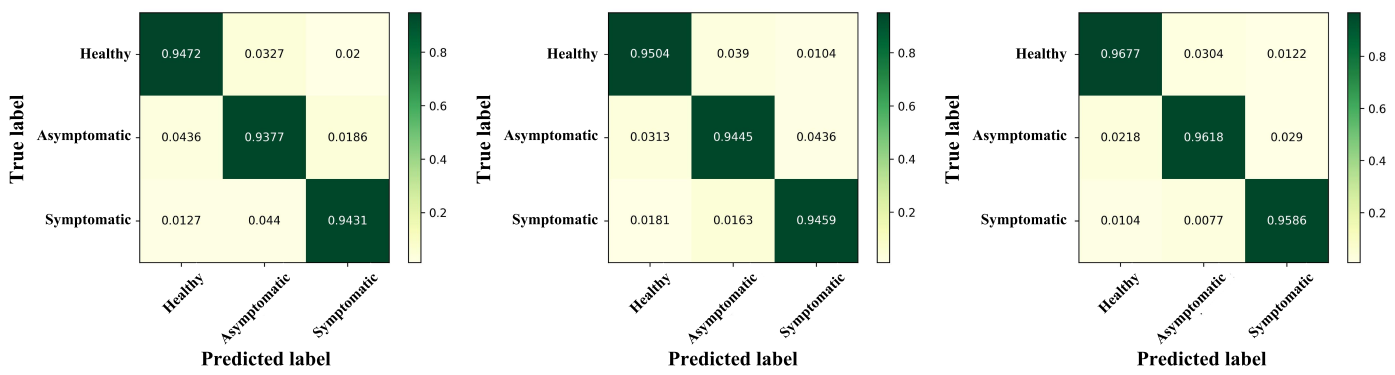 |
| --- |

(A) (B) (C)

**Figure S2.** Confusion matrix of DC^2^Net when different deformable convolution modules were applied: (A) deform2Dv1, (B) deform2Dv2, and (C) deform3D.

| 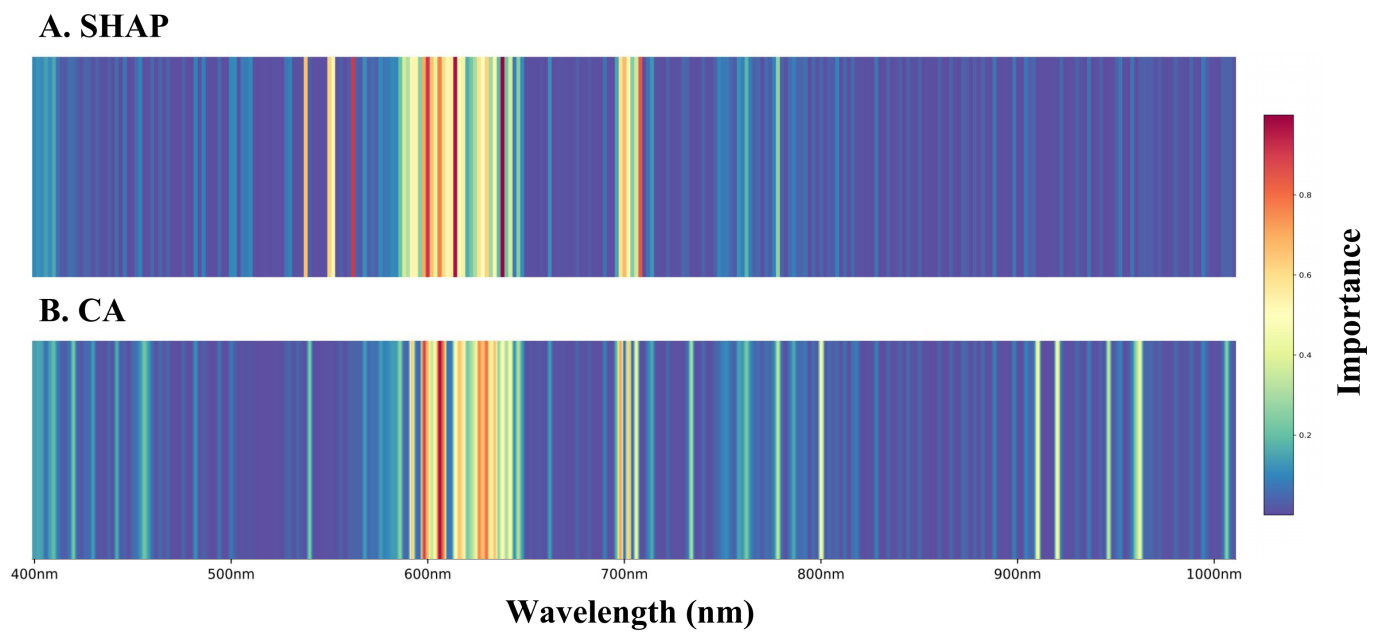 |
| --- |

**Figure S3.** Heat map of the importance value for all wavelengths. **Note:** The red color represents the most contributing feature wavelengths and the blue color represents the least contributing feature wavelengths. The spectral range was from 400 nm to 1000 nm with a total of 306 wavelengths. **Figure S3(A)** showed the interpretation of the DC^2^Net classification model using the SHAP method, while **Figure S3(B)** displayed the result generated by the channel attention method.

| 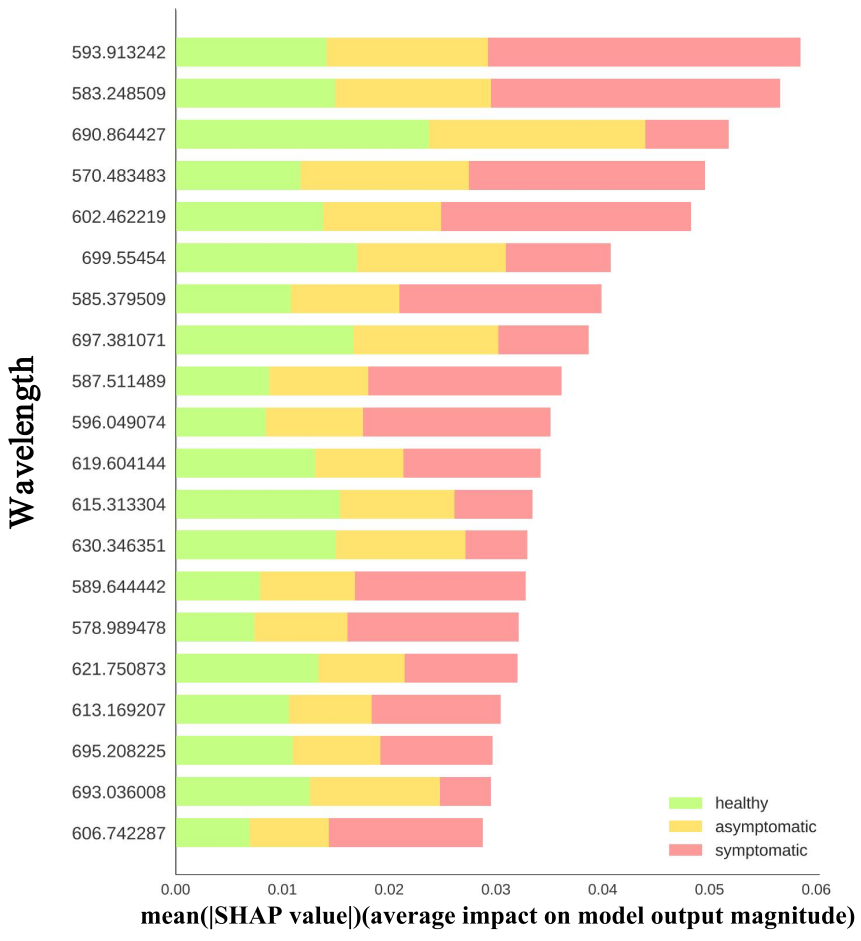 |
| --- |

**Figure S4.** Bar chart of the top 20 importance values for wavelengths in the three categories.

| 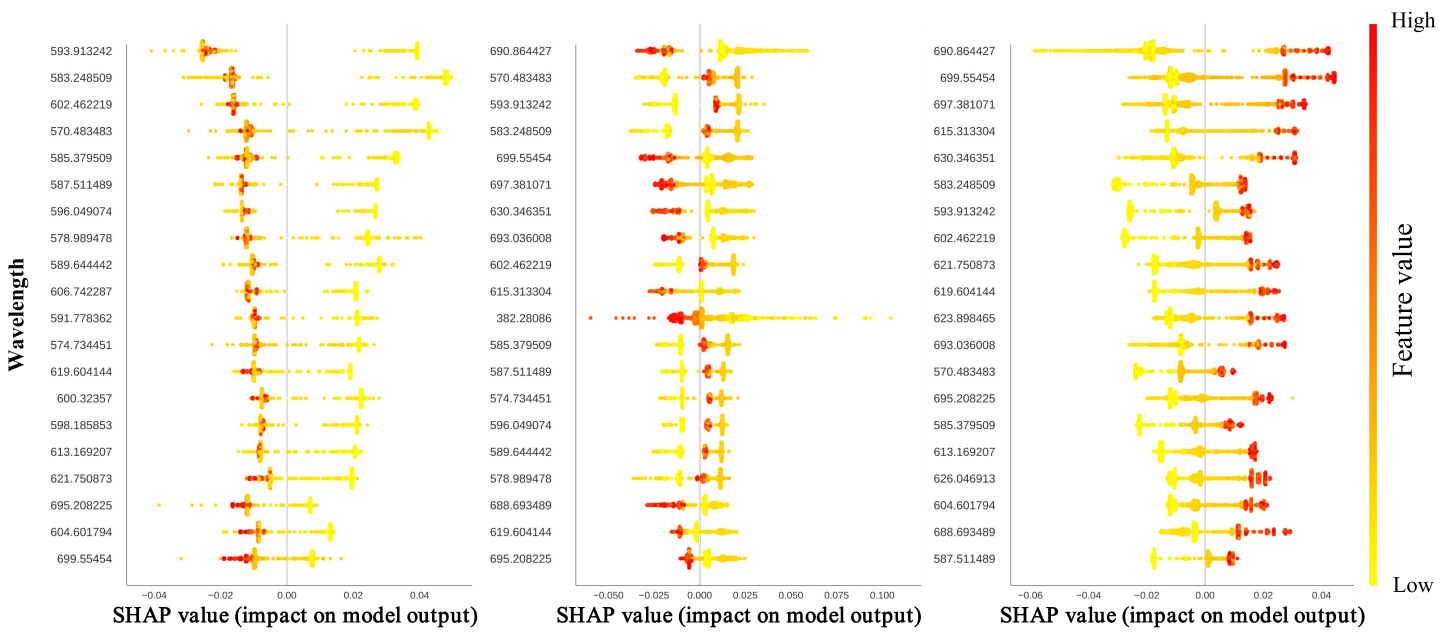 |
| --- |

(A) (B) (C)

**Figure S5.** Bee swarm plots of the top 20 importance values for wavelengths in the three categories. (A) Healthy, (B) Asymptomatic, and (C) Symptomatic. **Note:** Each dot represented a sample point, each row represented a screened wavelength, and the horizontal coordinate was the SHAP value. Red colors represented larger values and yellow color represented smaller values, with positive values indicating a positive impact on the predicted outcome and negative values indicating a negative impact on the predicted outcome.

| 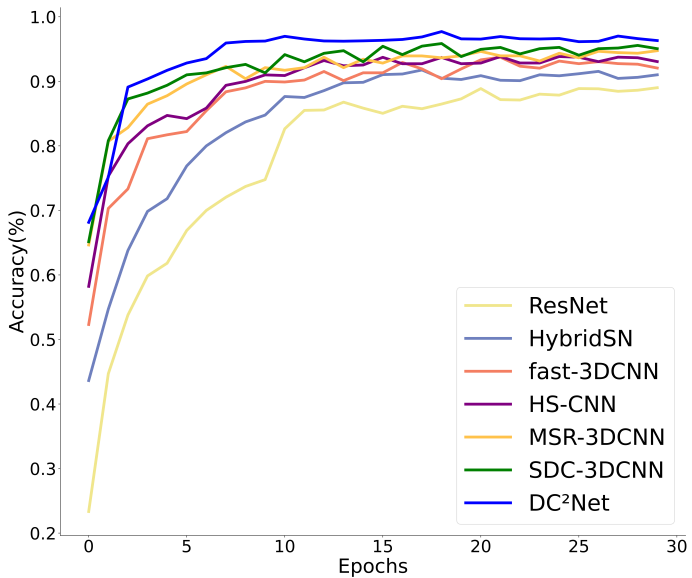 | 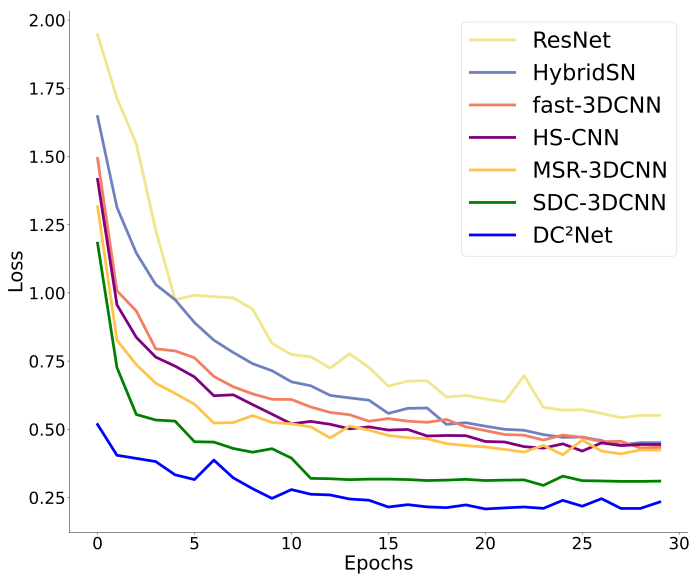 |
| --- | --- |
| (A) | (B) |

**Figure S6.** Performance comparison of SOTA models on author datasets: (A) accuracy plot, and (B) loss plot.

| 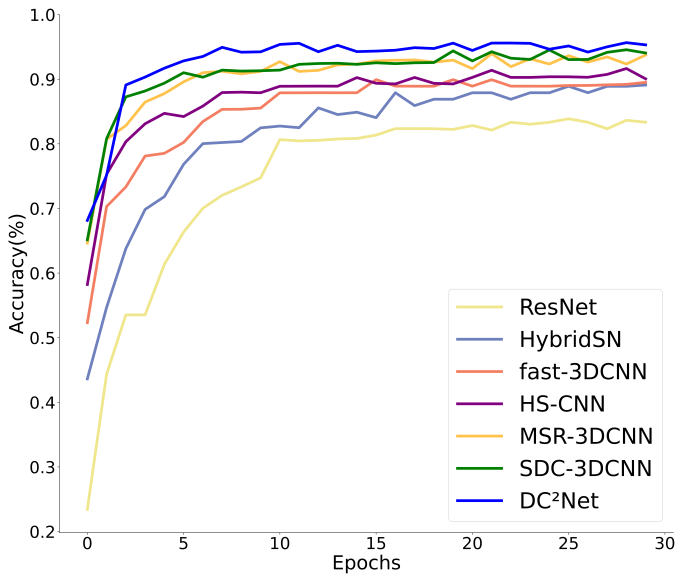 | 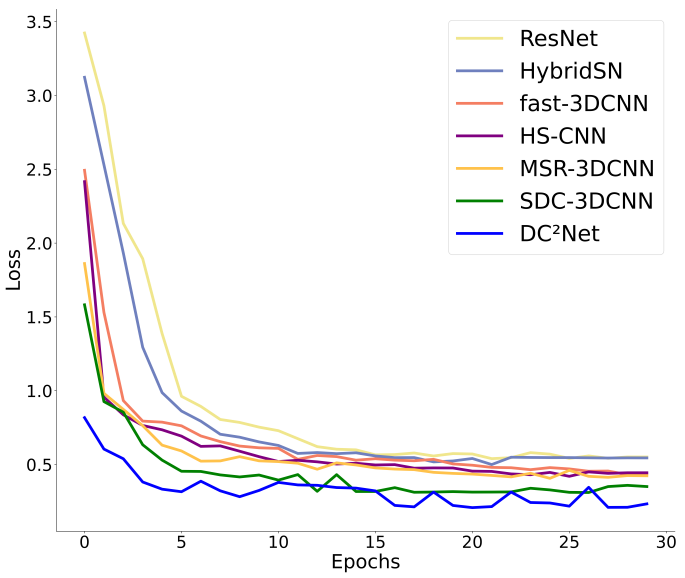 |
| --- | --- |
| (A) | (B) |

**Figure S7.** Performance comparison of SOTA models on public datasets: (A) accuracy plot, and (B) loss plot.
